# Innovative Therapeutic Strategies in the Treatment of Brain Metastases

**DOI:** 10.3390/ijms14012135

**Published:** 2013-01-22

**Authors:** Maria Caffo, Valeria Barresi, Gerardo Caruso, Mariano Cutugno, Giuseppe La Fata, Mario Venza, Concetta Alafaci, Francesco Tomasello

**Affiliations:** 1 Department of Neurosciences, School of Medicine, University of Messina, A.O.U. Policlinico “G. Martino”, via Consolare Valeria, 1, 98125 Messina, Italy; E-Mails: mcaffo@unime.it (M.C.); cutugnomariano@libero.it (M.C.); giuseppelf@alice.it (G.L.F.); mariovenza@gmail.com (M.V.); calafaci@unime.it (C.A.); ftomasel@unime.it (F.T.); 2 Department of Human Pathology, School of Medicine, University of Messina, A.O.U. Policlinico “G. Martino”, via Consolare Valeria, 1, 98125 Messina, Italy; E-Mail: vbarresi@unime.it

**Keywords:** angiogenesis, blood-brain barrier, brain metastases, cancer stem cells, microRNA, targeted therapy

## Abstract

Brain metastases (BM) are the most common intracranial tumors and their incidence is increasing. Untreated brain metastases are associated with a poor prognosis and a poor performance status. Metastasis development involves the migration of a cancer cell from the bulk tumor into the surrounding tissue, extravasation from the blood into tissue elsewhere in the body, and formation of a secondary tumor. In the recent past, important results have been obtained in the management of patients affected by BM, using surgery, radiation therapy, or both. Conventional chemotherapies have generally produced disappointing results, possibly due to their limited ability to penetrate the blood–brain barrier. The advent of new technologies has led to the discovery of novel molecules and pathways that have better depicted the metastatic process. Targeted therapies such as bevacizumab, erlotinib, gefitinib, sunitinib and sorafenib, are all licensed and have demonstrated improved survival in patients with metastatic disease. In this review, we will report current data on targeted therapies. A brief review about brain metastatic process will be also presented.

## 1. Introduction

Metastasis, the spread of cancer from the site of primary tumor growth to distant organs, is a leading cause of cancer morbidity and mortality. The metastatic process requires invasion from the primary tumor, intravasation, survival, arrest, extravasation of the circulatory system, and colonization of a distant site. Metastatic brain tumors are the most common intracranial neoplasm in adults, and although the exact incidence is unknown, it has been estimated that they can occur in up to 30% of patients [1]. The incidence of brain colonization strongly depends on the tumor type and, in some cancers, also on the molecular subtype. In adults, brain metastases (BM) commonly arise from primary tumors of the lung (40%–50%), breast (15%–25%), melanoma (5%–20%), renal and gastrointestinal tract (4%–6%) [2]. The incidence of BM is rising. The reasons are likely multifactorial and include an increasing incidence of lung cancer associated with tobacco use, generally longer survival times of cancer patients, and increased utilization of cranial magnetic resonance imaging (MRI) in the upfront staging and follow-up. Furthermore, the advent of novel therapeutic compounds with good anti-neoplastic activity but inadequate penetration via the blood–brain barrier (BBB) may also contribute to an increase of BM.

Current therapeutic approaches for BM include surgery, whole-brain radiation therapy (WBRT), stereotactic radiosurgery (SRS), chemotherapy, or a combination of these therapies. Survival for patients with BM treated with WBRT typically ranges from 4 to 6 months, but can be as long as 12–24 months for some patients [3]. Strong positive prognostic factors include good functional status, age <65 years, no sites of metastasis outside of the CNS, controlled primary tumor, the presence of a single BM, and long interval from primary diagnosis to brain relapse [4]. Randomized clinical trials have shown that surgery or SRS combined with WBRT improves overall survival compared with WBRT alone in patients with a single metastasis in the brain [5–7]. The combination of radiotherapy and chemotherapy improves response rate and/or progression-free survival (PFS) in some studies [8,9], but not overall survival (OS) [10]. Systemic antineoplastic therapy has shown limited or no efficacy in BM, although comprehensive studies are almost lacking. However, recent studies have better cleared some features of the BM process such as tumor cell migration, invasion and metastasis. These new researchers support the development of new therapeutic agents that could inhibit brain metastasis formation.

## 2. Brain Metastases Development

Metastasis of cancer cells is a highly selective, non-random process consisting of a series of linked sequential events. According to the seed and soil concept, brain colonization is driven by a specific affinity of certain tumor cells for the milieu of certain organs. The specific reasons for the variable brain-tropism among tumor types remain unclear, although a relation to molecular factors rather than simply to the anatomy of blood perfusion has been postulated [2]. The initial stages of metastasis involve the dispersal and migration of individual tumor cells away from a primary tumor. This process involves modulation of cell–cell interactions and cell-substrate adhesions, as well as migration and invasion into the surrounding extracellular environment. The tumor cells must then migrate through vascular endothelial cells or lymphatic vessels to enter the circulation (intravasation process). An immature neovascular system, high interstitial pressure, and the close proximity of cancer cells to blood vessels are factors favoring tumor cell intravasation. Once in the circulation, the intravascular tumor cells are subject to non-specific mechanical forces, such as hemodynamic turbulence, which might cause mechanical destruction of the cells, either before or during the extravasation process. However, many tumor cells successfully escape from the circulation and reach secondary sites (extravasation process). During extravasation, tumor cells encounter different microenvironments with a significant adaptation to allow survival and colonization of secondary tissues. Thus, individual cells and/or micro-metastases can lie dormant for periods of months or even years before developing into macroscopic metastases. All these events are driven by gene products of metastatic cells, and by direct cell-to-cell and paracrine interactions between cancer cells and various stromal cells, both in the primary and in the metastatic tumor microenvironments [11–14]. During disease progression, cancer cells activate local stromal cells, including resident fibroblasts and macrophages, and attract to the primary tumor circulating monocytes and platelets. In turn, the reactive stroma-associated cytokines, chemokines, growth factors and matrix metalloproteinases mediate attraction of bone marrow-derived stem and progenitor cells to the microenvironment of the primary tumor. These active agents also mediate angiogenesis, degradation of basement membrane barriers and other extracellular matrix components, as well as detachment, motility and migration of cells from the primary cancer, thus promoting local tumor growth and invasion [11–13,15].

### 2.1. Invasion

Local brain invasion is a multifaceted process of tightly controlled mechanisms including cell motility, adhesion, and enzymatic remodeling of the extracellular matrix (ECM). Brain invasion requires paracrine interactions between brain stromal, endothelial cells and invading metastatic cells. Proteolytic degradation of the ECM is thought to aid tumor invasion by clearing a pathway for the invading tumor cells. The E-cadherin–catenin complex is a prime mediator of cell–cell adhesion and is crucial for intercellular adhesiveness, and the maintenance of normal and malignant tissue architecture. Reduced expression of this complex has been associated with tumor invasion, metastasis, and an unfavorable prognosis [16]. Once metastatic cancer cells enter the brain circulation, they might arrest in sites of slow flow within the capillary bed at vascular branch points, which is then followed by early changes in the brain microenvironment. The brain vascular endothelial cells and the stromal cells such as fibroblasts associated with the primary tumor are involved in metastatic nodules in the brain. These co-disseminating stromal cells provide survival and proliferative advantages to the tumor cells and facilitate early colonization of metastatic foci.

Adhesion to the ECM is fundamental in the invasion phenomenon. The integrins are the predominant family of cell adhesion molecules that mediate ECM and basement membrane adhesions during metastasis. Integrins are heterodimeric transmembrane cell surface receptors that regulate cell adhesion, migration, differentiation, proliferation, and survival during physiological and pathological conditions. Upon ligation to extracellular ligands, integrins activate downstream signaling pathways in concert with growth factor receptors, including platelet-derived growth factor receptor (PDGFR), epidermal growth factor receptor (EGFR) and vascular endothelium growth factor receptor (VEGFR). The integrins, the ECM and the microfilaments inside the cell are connected by proteins as talin, paxillin, and alpha-actinin. These act by regulating the kinase such as focal adhesion kinase (FAK) and the family of Src kinases to phosphorylated substrates such as the p130CAS or recruiting signal adapters such as Crk. Interestingly, the tumor suppressor gene PTEN encodes for a cytoplasmic tyrosine phosphatase that dephophorylates inositol lipids generated by PI 3-kinase, and also other components of focal contacts including FAK and Shc. Thus, PTEN has been shown to suppress integrin-mediated signaling when overexpressed, and, conversely, loss of PTEN function may contribute to tumorigenesis and metastasis through modulation of integrin function. Expression of α_ν_β_3_, a vitronectin-binding integrin, has been associated with increased metastatic potential presumably through its ability to allow the cancer cells to interact with platelets in the blood stream, thereby arresting the cancer cells in the blood flow [17]. Yoshimasu *et al*. developed a brain-seeking cell line from EBC-1 lung cancer cells and found that the EBC-1/brain cells were more adherent to fibronectin, type I collagen, and laminin than the parental EBC-1 cell line [18]. A recent study shows that tumor cell integrin α_ν_β_3_ activation strongly promotes metastatic growth in the brain by enabling tumor cells to attract blood vessels independently of hypoxia [19]. The specific ability of activated tumor cell integrin α_ν_β_3_ to enhance angiogenesis depends on the tissue microenvironment. Organ-dependent differences in growth factors, chemokines, cytokines, and matrix proteins, as well as stromal and immune components, may distinctly affect tumor cell growth and endothelial behavior in the mammary fat pad *versus* the brain and may synergize with (brain) or antagonize growth-promoting functions of activated tumor cell α_ν_β_3_ [19]. The mechanism through which activated α_ν_β_3_ supports brain metastatic growth is based on elevated expression of vascular endothelial growth factor because of inhibition of translational repressor 4E-BP1, resulting in efficient tumor angiogenesis under normoxic conditions. This function prevents development of hypoxia, associated tumor cell apoptosis, and retardation of lesion growth [19].

Extracellular proteolytic enzymes are critical for the invasive properties of malignant neoplasms. These also include the matrix metalloproteinases (MMPs) and the urokinase-dependent plasminogen-activating cascade.

MMPs comprise a large family of zinc-dependent endoproteinases, collectively capable of degrading all ECM components. The proteolytic activities of MMPs influence essential cellular processes like cell proliferation, migration and adhesion, as well as many fundamental physiological events such as angiogenesis, bone development, wound healing, and uterine and mammary involution. Once active, MMPs are regulated by interactions with endogenous inhibitors including α2-macroglobulin, thrombospondin-2, tissue inhibitors of metallo-proteinases (TIMPs) and reversion-inducing cysteine-rich protein with kazal motifs (RECK) [20]. MMP activity has been correlated with invasiveness, metastasis, and poor prognosis in metastatic tumors. Jaalinoja *et al.* reported that all metastatic brain tumors were positive for MMP-2 [21]. Arnold *et al.* reported that MMP-9 was upregulated in all BM [22]. A second proteolytic system that interfaces with MMPs is the urokinase pathway of plasminogen activation. This system includes urokinase (urokinase-type plasminogen activator, uPA), the urokinase receptor (uPAR), and plasminogen. Thus, increased activation and or expression of uPA, uPAR and/or PAI-1 has been associated with tumor progression and poor prognosis in patients with various malignant tumors, including breast, lung, kidney, ovary, cervix, colon, stomach and soft tissue cancers. Activated uPA converts plasminogen into plasmin, a serine protease that promotes cellular migration by the degradation of ECM proteins, activation of other matrix proteases and activation of cell surface receptors.

### 2.2. Angiogenesis

The growth and proliferation of a metastatic tumors is dependent on the establishment of an adequate blood supply [23,24]. Different mechanisms have been evaluated, including the formation of new blood vessels (vasculogenesis), the utilization of existing blood vessels (co-option), and the sprouting from existing blood vessels (angiogenesis). A tumor can also recruits blood vessels via: vessel remodeling and expansion by the insertion of interstitial tissue columns into the lumen of pre-existing vessels (intussusception), cancer cells lining blood vessels (vasculogenic mimicry), cancer cells that transdifferentiate into endothelial cells, [25], and cancer stem-like cells that form an inner lining of blood vessels in the brain [26,27].

Kusters *et al*. using a melanoma cell line injected into the internal carotid artery, showed that a brain metastasis could grow up to 3 mm modulating the pre-existing blood vessels [28]. Carbonell *et al.* used several breast and melanoma cell lines evidenced that the growth of micrometastasis in the brain was dependent on the co-option mechanism and that the co-option process is an active adhesive mechanism between the tumor cells and the exterior of the blood vessels [29]. They showed, also, that β_1_ integrin expressed by the tumor cell lines is the key component of co-option through its specific interaction with the vascular basement membrane [29]. Mel57 human melanoma cells produced little endogenous VEGF but established infiltrative BM in mice by co-opting existing peritumoral vessels, thus indicating that the preexisting vasculature can contribute to metastatic growth [30]. Kim *et al.* reported an increase in blood vessel density, as well as vascular remodeling. In human MDAMB-231 breast cancer cells isolated from the brain, a significant VEGF-A expression and a higher microvessel density was demonstrated [31]. BM from murine melanoma, murine fibrosarcoma, human lung carcinoma, and human colon carcinoma have a lower microvascular density than the surrounding normal brain parenchyma, and they all contain dilated blood vessels with large lumens [32]. The early steps of angiogenesis include degradation of the endothelial basement membrane and surrounding ECM, and directed migration of endothelial cells into surrounding stroma toward angiogenic stimuli. The balance between inducers and inhibitors of angiogenesis is critical in determining the generation or not of new vessels. Although a plethora of molecules can act as inducers of angiogenesis such as acidic fibroblast growth factor (aFGF), basic fibroblast growth factor (bFGF), transforming growth factor alpha and beta (TGF-α and -β), tumor necrosis factor alpha (TNFα) and interleukin-8 (IL-8), the major growth factors specific for vascular endothelium include members of the VEGF and angiopoietin families.

Angiogenesis may be quantified in tissues by the assessment of the microvessel density (MVD), which reflects the number of vessels/mm^2^ [33]. The MVD may be assessed by highlighting the vessels present in tissue section through standard immunohistochemistry against endothelial markers, such as Factor VIII, CD31, CD34 and endoglin. Following the use of antibodies against pan-endothelial markers (Factor VIII, CD31, CD34), all the vessels present in the histological section are stained, with no distinction between pre-existing and newly formed vessels [33]. In contrast, staining for endoglin (CD105), a 180-kDa transmembrane homodimeric glycoprotein that belongs to the TGF receptor complex allows a more specific detection of the vessels related to neoangiogenic process [33,34]. Thus MVD evaluated by antiendoglin antibody may be used as a tool to define whether metastatic tumors are to be considered “angiogenic-dependent” or not and, consequently, whether they are suitable targets for angiogenesis-blocking therapies. The only study investigating MVD in brain metastases tissue [35] has shown a significantly lower neo-angiogenesis in CNS metastases from malignant melanoma than in those from breast cancer, suggesting a potential low efficacy of antiangiogenic therapy in melanoma metastatic to CNS. Vasculogenic mimicry (VM) represents the *de novo* generation of blood vessels without the participation of endothelial cells and independent of angiogenesis. In VM vascular channels with flowing blood plasma and red blood cells are composed of a basement membrane and formed by the tumor cells, in the absence of endothelial cells [36], and connected to the host blood vessels. In a recent study of human melanoma biopsies and melanoma xenografts, it has been observed that VM and leaky vessels are a direct contributor to hematogenous spread. The low percentage of tumor cells directly contributing to the capillary network is interesting concerning tumor cell plasticity and tumor cell niche aspects [37]. Though at present no data exist on the presence of VM in BM, this may represent an additional mechanism of resistance to antiangiogenic therapies, which deserves further investigation.

### 2.3. Molecular Features

Biological studies, in the past years, have evaluated important molecular interactions between tumor cells and stromal environment that regulate the tissue specificity of metastasis formation. Molecular factors may be organ specific and influence the tumor cells with regard to gene and protein expression, growth dynamics, and responsiveness to treatment [38]. The discovery of the silence of tumor-related genes in the metastatic process came to shed new data in the tumor invasion phenomenon. The metastasis suppressor genes (MSG) (Table 1) suppress the formation of spontaneous, macroscopic metastases without affecting the growth rate of the primary tumor [39].

KAI1 (CD82) encodes a protein that belongs to the tetraspanin superfamily. This family of proteins regulates adhesion, migration, growth, and differentiation. Downregulation of metastasis is observed in cancer of the colon, liver, esophagus, pancreas, lung, bladder, ovaries, cervix, and breast when this protein is overexpressed. KAI1 interacts with E-cadherin, β1 integrins, and EGFR. E-cadherin, when it is expressed with KAI1, might increase activity in suppressing metastatic tumor spread [40].

Nm23-H1, found in breast cancer and melanoma, encodes a nucleotide diphosphate protein kinase that appears to regulate cell growth by interacting with Menin (a tumor suppressor gene product), cell centrosome by inhibiting Rad (a growth promoting Ras-related GTPase), by binding to DNA to activate transcription of the Rb2 cell cycle inhibitor, and repressing transcription of platelet-derived growth factors [41]. Similarly, Nm23-H2 reduces metastatic potential and cell motility. Probably, this gene could regulate the reorganization of the actin cytoskeleton and encode signaling proteins that stimulate changes in adhesion status [42].

Mitogen-activated protein kinase kinase 4 (MKK4) is involved in signal transduction between MEKK1, protein kinase/JNK1, and p38 MAPK. The overexpression of the MKK4 gene results in reduced of spontaneous metastases by 70%, whereas it had no effect on primary tumor growth [43].

CD44 codes for a transmembrane protein involved in cell adhesion by binding to specific extracellular matrix components. CD44 may control the adhesion processes of circulating cancer cells to endothelium at the secondary site with the help of hyaluronate matrix ligand or by its cytoplasmic attachments to actin-associated proteins of the merlin/ezrin/radixin/moesin family [44].

KISS-1 is a gene which encodes metastin (fragment of KISS-1), and it is expressed primarily in melanoma and breast cancer cells. Metastin, a ligand of the orphan G protein coupled receptor hOT7T175, inhibits chemotaxis, invasion, and spreading, monolayer growth [45].

Brms1 is expressed primarily in melanoma and breast cancer cells. It restores the normal gap junction phenotype, which in turn maintains the communication between cells within the primary tumor, but reduces seed and soil communication at the secondary sites [46].

RhoGD12 (guanine nucleotide binding protein) has been designated as a MSG. Increased RhoGD12 RNA expression in a bladder carcinoma cell line has been associated with decreased metastatic potential, and gene expression profiling of 105 clinical tumor specimens from multiple organ sites recorded an inverse correlation with the invasive potential of these tumors [47].

The PTEN phosphatase and tensin homolog deleted on chromosome 10 or MMAC1 (mutated in multiple advanced cancers) tyrosine phosphatase was found to be mutated in a variety of cancers, including those of the brain, breast and prostate. Tamura *et al.* demonstrated that overexpression of PTEN/MMAC1 inhibited cell migration, whereas antisense PTEN/MMAC1 enhanced migration [48]. Hahn *et al.* evidenced that, in BM from various primary tumors, there was a low frequency of PTEN/MMAC1 mutation detection (14%), indicating that one or more additional tumor suppressor genes may be present on chromosome 10 [49].

Loss of the Nf2 tumor suppressor gene, which encodes the Nf2 membrane/cytoskeleton linker protein, has also been implicated in metastasis, predominantly through the use of Nf2 knockout mice that developed various metastatic malignancies [50]. Loss of p53 function has been implicated in tumor progression not only due to its effect on angiogenesis but also because p53 is important for maintaining genetic stability, such that its loss results in an accumulation of genetic changes leading to the metastatic phenotype [51].

*N*-cadherin is involved in multiple processes including inducing invasion, migration, promoting survival of cancer cells, regulating adhesion and neurite outgrowth. Nguyen *et al.* developed a T-cell factor 4 (TCF4) signature prognostic for lung metastasis to multiple organs. Validation of these findings have identified three genes from the TCF4 signature that were highly correlated with metastatic development—LEF1, HOXB9, and BMP4. Confirming that LEF1 and HOXB9 are involved in metastasis, overexpression of the two genes led to an increase in bone and BM whereas knockdown of each gene decreased metastatic incidence [52]. Bos *et al.* derived brain-seeking cells from the triple-negative human MDA-MB-231 cell line and from the tumor of an ER patient (CN34), found 243 genes differentially expressed between the BM and parental cell lines. Of those, the expression of 17 genes was correlated with brain relapse. Both the breast and lung sets also expressed an EGFR ligand indicating that the EGFR pathway may play an important role in cancer metastasis to the brain [53]. To identify genes that were responsible for brain specificity, the gene expression patterns of organ-tropic MDA-MB-231 bone-, lung-, and brain-seeking cells and CN34 brain-seeking cells were determined. Twenty-six upregulated genes were identified in the brain-seeking cells, including ST6GALNAC5, an a-2,6-sialyltransferase whose expression is typically found in the brain [53].

A variety of oncogenes have been implicated in the metastatic process. Activating mutations in the Ras family of small GTP-binding proteins are frequently observed in various human tumors and result in a metastatic phenotype upon expression in a variety of cell types. In addition to Ras, ectopic expression of other oncogenes includes the serine/threonine kinases Mos and Raf, as well as the tyrosine kinases Src, Fms and Fes. Using effector domain mutants of V12-HRas that are impaired for their abilities to activate defined downstream targets, it has been demonstrated that activation of the Raf-mitogen activated protein kinase (MAPK) 1/2 pathway is key to the development of experimental lung metastases [54].

The oncoprotein Stat3 is constitutively activated in cancer cells in various types of human cancer. The Stat3 signaling pathway may impact tumor metastasis via regulation of several types in this process. Stat3 is activated by many cytokines and growth factors, including epidermal growth factor and interleukin-6, as well as by oncogenic proteins, such as Src and Ras. Conversely, Stat3 activation is negatively regulated by several proteins, including the suppressor of cytokine signaling (SOCS)-1. Prior studies have suggested that activated Stat3 promotes tumor growth and metastases, presumably through its critical role in the expression of many genes key to regulation of multiple aspects of tumor cell survival, growth, angiogenesis, and evasion of immune surveillance, such as cyclin D1, MMP-2, VEGF, and 10-kDa IFN-g-induced protein [55]. Recently, it has been demonstrated that Stat3 activation transcriptionally represses caveolin-1 expression and promotes breast cancer invasion and brain metastases [56]. The role of Stat3 in the promotion of brain metastasis has been, also, shown in A375 human melanoma cell line [57]. Genes in which expression was altered by increased Stat3 expression included several involved in invasion and angiogenesis, including MMP-2, bFGF, and VEGF, therebysuggesting that Stat3 may serve multiple prometastatic roles.

### 2.4. MicroRNA

MicroRNAs (miRNAs) are a group of short (21–23) nucleotides that control the expression of many target genes at the posttranscriptional level. They are aberrantly expressed in many types of cancer, and play a major role in regulating a variety of targets and pathways, which making them a useful tool for early detection of disease, management, and prognosis. MicroRNAs can function either as tumor suppressors or as oncogenes and initiate tumor growth, invasion, metastases, the process of epithelial to mesenchymal transition (EMT), as well as regulate the overall stemness of cancer cells [58]. One single miRNA can target several oncogenic proteins and the opportunity to simultaneously repress several oncogenes appears very interesting in the anticancer therapy, while limiting possible side effects. Hence therapeutic strategies can include direct tumor suppressive effects, antiangiogenic effects, antimetastatic effects, suppression of immune evasion of tumors, or sensitization of tumor cells to traditional anticancer treatments such as radiotherapy or chemotherapy. On the other hand, miRNA-based therapies can be based on the development of miRNA-mimicking compounds to increase the cellular concentration of any given miRNA or, on the contrary, of miRNA antagonists to reduce their level. Although the total number of micro-RNAs remains controversial and the roles of specific miRNAs are only beginning to be defined, high throughput miRNA expression analyses indicate that these species represent promising candidates for clinical tumor cell markers.

Rising evaluation has demonstrated that one of the first steps in tumor progression may be mediated through the acquisition of EMT phenotype of cancer cells, which is a process by which epithelial cells lose their cell-to-cell contacts and, subsequently, attain characteristics of mesenchymal phenotype. These cells detach from the primary tumor site and enter into vascular district which is believed to be responsible for tumor cell metastasis [59,60]. The acquisition of EMT phenotype could be regulated by deregulation in the expression of miRNAs in the context of metastasis. In a recent study, a new diagnostic tool to aid in the differentiation between primary and secondary neoplasms of the CNS has been demonstrated. In this research, a combination of two specific miRNAs which serves as a novel brain primary tumor biomarker has been identified. Specifically, the authors have been found that hsa-miR-92b and hsa-miR-9/9* are very significantly and strongly overexpressed in samples of primary brain tumors, but not in samples of metastatic tumors to the brain. The combined expression levels of hsa-miR-92b and hsa-miR-9/9* allow discrimination between brain primary tumors and metastases located in the brain with very high accuracy, and thus represent a potential biomarker for the identification of brain primary tumors [61]. A link between miR-1258 and heparanase, which represents a novel mechanism of endogenous regulation of this molecule known to support metastasis, has been recently identified [62]. It has been reported that heparanase regulates the expression of many molecules involved in angiogenesis and metastasis, including MMP-9, COX2, and EGFR [63]. The authors demonstrated that treatment of miR-1258 resulted in changes of MMP-9, COX2, and EGFR protein expression. Moreover, by transducing lentiviral vectors expressing miR-1258 HPSE inhibition by miR-1258 decreased MMP-9, COX2 protein levels, and phosphorylation of Akt and EGFR [62]. A recent experimental study revealed the expression profiling of BM from colorectal cancer [64]. The results indicated that an overexpression of miR-145, miR-1, miR-146a, miR-576-5p, miR-126*, HS_287, miR-28-5p, miR-143, miR-199b-5p, miR-199a-5p, miR-10b, miR-22, miR-133b, miR-145*, miR-199a, miR-133a, miR-125b, and a downregulation of miR-31 and HS_170, occurred in brain metastatic carcinomas. A recent study has demonstrated that miR-146a is virtually absent from BM and can suppress their metastatic potential including their migratory and invasive activities associated with upregulation of β-catenin and downregulation of hnRNPC [65]. Brain-trophic metastatic MDA-MB-435-LvBr2 (LvBr2) cells via left ventricle injection of MDA-MB-435 cells into immunodeficiency (NOD/SCID) mice were isolated. LvBr2 cells expressed lower β-catenin levels and higher heterogeneous nuclear ribonucleoprotein C1/C2 (hnRNPC) levels. MicroRNA-146a was almost undetectable in LvBr2 cells and highly expressed in the parental cells. Overexpression of miR-146a increased β-catenin expression and suppressed the migratory and invasive activity of LvBr2 cells. The miR-146a-elicited decrease in hnRNPC in turn lowered the expression of MMP-1, uPA, and uPAR and inhibited the migratory and invasive activity of LvBr2 cells. MicroRNA-328 is inversely correlated with ABCG2 expression in glioblastoma CSCs [66] and is also associated with chemoresistance. Modulation of either miR-328 or ABCG2 protein expression increased the efficacy of chemotherapeutic agents [66]. Arora *et al.* conducted miRNA microarray profiling on samples from seven non-small cell lung cancer (NSCLC) patients with BM and five without BM. It was determined to be overexpressed in patients with BM, and appears to play a role in establishing migratory potential of NSCLC cells, in particular through the deregulation of PRKCA gene [67].

### 2.5. Cancer Stem Cells

Cancer stem cells (CSCs) and de-differentiated cancer cells are capable of using several intracellular pathways that are analogous to those used by normal stem/progenitor cells and their progeny during development, despite the dysregulation of many of their biological functions. During the process of de-differentiation, there is deactivation of repressive mechanisms of developmental transcription factors, and activation of dormant intracellular signaling pathways which are ordinarily expressed during development by normal stem cells [68–70]. The ability of CSCs to induce angiogenesis, to migrate, to invade tissues and blood vessels and to infiltrate and colonize distant tissues is in part mediated by cellular pathways which are expressed ordinarily by normal tissue-specific stem/progenitor cells. Three of these signaling pathways which are particularly important in carcinogenesis are the interaction between chemokine stromal derived factor-1 (SDF-1) and its chemokine receptor CXCR4, the EMT pathway and the Wnt pathway [11,12,15,71–73].

Neural stem cells are the origins of neurons and glia, and generate all the differentiated neural cells of the mammalian CNS via the formation of intermediate precursors. Regulation of neural stem cell number during CNS development and in adult life is associated with rigorous control. Failure in this regulation may lead to, e.g., brain malformation, impaired learning and memory, or tumor development. Detailed molecular characterization together with novel stem cell-like glioma cell models that reflect the original tumor gives opportunities for research into new therapies. The identification of these cells, in addition to the pathways that regulate their maintenance, may allow selective targeting of the core population of tumor promoting cells.

Pommier *et al.* demonstrated the presence of subpopulations of stem cells within breast cancers that express genes responsible for breast proliferation and cellular proliferation. In this experimental study, the cells that showed the highest level of tumorigenicity, evidence the multipotent capability of the CD49f^+^ CD24^−^ cells [74]. Guo *et al.* evidenced that GI-101A breast cancer cells possess the capacity of growth in multiple target organs with an intrinsic tropism for brain tissue [75]. In an experimental BM model, intravenously administered human neural stem cells line (F3.CD-TK) expressing the dual suicide genes cytosine deaminase (CD) showed remarkable bystander killer effect CD and herpes simplex virus thymidine kinase (TK) [76]. F3 cells migrated near lung cancer metastatic lesions, which were induced by the injection of lung cancer cells via the intracarotid artery. More important, F3.CD-TK cells in the presence of prodrugs 5-fluorocytosine and ganciclovir decreased tumor size and considerably prolonged animal survival. The neural stem cells capacity to target multiple foci of brain metastases in a syngeneic experimental melanoma model was, recently, evaluated [77]. In this experimental model, animals with established melanoma brain metastasis received intracranial implantation of cytosine deaminase-expressing neural stem cells followed by systemic 5-fluorocytosine treatment, resulting in a significant (71%) reduction in tumor burden. In a novel experimental study, the efficacy of brain transplantation of human NSCs, encoding the suicide enzyme carboxyl esterase combined with systemic administration of the prodrug CPT-11 (irinotecan), has been evaluated [78]. NSCs expressing rabbit carboxyl esterase migrated selectively into the brain metastases. Moreover, a significant inhibition of the MDA-MB-435 cells growth was also obtained.

Overexpression of B lymphoma Moloney murine leukemia virus insertion region-1 (BMI1), a transcription repressor that operates in stem cell maintenance and oncogenesis through inhibition of INK4A/ARF tumor suppressor locus, has been linked with increased incidence of metastasis in human gastric and breast cancer, as well as melanoma and other cancer types. Additionally, BMI1 has recently been associated with a stem cell-like 11 gene expression microarray signature predictive of a short interval to disease recurrence following therapy, increased likelihood of metastatic disease, and poor response to therapy in multiple types of human cancer, including prostate, lung, ovarian, urinary bladder, lymphoma, mesothelioma, glioma, acute myeloid leukemia, and breast cancer [79]. Hoenerhoff *et al.* have demonstrated that BMI1 in conjunction with H-RAS in human mammary epithelial cells (HMECs) results in an aggressive phenotype that includes spontaneous metastasis to liver and spleen, as well as novel metastasis to brain [80]. Notch proteins are a family of four transmembrane, heterodimeric receptors (Notch1IC-Notch4IC), with five known ligands (Delta-like1, Delta-like3, Delta-like4, Jagged1 and Jagged2). Altered Notch signaling has been observed in many human cancers, including endometrial cancer, colon cancer and lung cancer. Nam *et al.* reported that a MDA-MB-435 carcinoma cell line selected for metastatic growth in the brain exhibited upregulation of the Notch pathway as compared to the parental cell line, and that the commercial γ-secretase inhibitor DAPT and RNA interference-mediated knockdown of Notch1 inhibited tumor cell migration and invasion *in vitro* [81]. McGowan *et al.* have shown that Notch signaling plays a significant role in the formation of brain metastases from breast cancer, partially due to its role in maintaining CSC (CD44hi/CD24lo) putative cancer stem-like cells. Inhibition of Notch1 *in vitro* resulted in decreased cell proliferation and invasion, and reduced expression of Notch 1–4 mRNA [82].

## 3. Blood–Brain Barrier (BBB)

The brain is one of the least accessible organs for the delivery of pharmacological compounds [83–85]. Specific interfaces tightly regulate the exchange between the peripheral blood circulation and the cerebrospinal fluid (CSF). These barriers are the choroid plexus epithelium (blood–ventricular CSF), the arachnoid epithelium (blood–subarachnoid CSF) and the blood–brain interstitial fluid. Their function is to maintain a constant environment inside the brain, by strictly regulating the composition of the cerebral extra-cellular fluid and to protect the brain against potentially toxic substances. The microvessel endothelial cells that form the BBB display important morphological characteristics such as the presence of tight junctions (TJs) and adherent junctions (AJs), the absence of fenestrations, and a low pinocytic activity. TJs are located on the apical region of endothelial cells and are structurally formed by a complex network made of a series of parallel, interconnected, transmembrane and cytoplasmatic strands of proteins [86]. TJs consist of three integral membrane proteins, namely, claudin, occludin, and junction adhesion molecules, and a number of cytoplasmic accessory proteins. The high level of integrity of TJs is reflected by the high electrical resistance of the BBB (1500–2000 Ω cm^2^), which depends on a proper extracellular Ca^2+^ ion concentration. Cytoplasmic proteins link membrane proteins to actin, which is the primary cytoskeleton protein for the maintenance of structural and functional integrity of the endothelium. AJs are located below the TJs in the basal region of the lateral plasma membrane. They are composed of transmembrane glycoproteins (cadherins) linked to the cytoskeleton by cytoplasmatic proteins, thus providing additional tightening structure between the adjacent endothelial cells at the BBB. The transport of solutes and other compounds across the BBB is strictly constrained through both physical tight junctions and adherent junctions.

There are different mechanisms by which solutes move across these barriers. The transport may occur due to diffusion, either simple diffusion or facilitated transport across aqueous channels. Passive diffusion is a concentration gradient-dependent process that allows molecules to move across cellular membranes between cells (paracellular way) or across cells (transcellular way) down their electrochemical gradient without the requirement of metabolic energy. Small lipid soluble substances like alcohol and steroid hormones penetrate transcellularly by dissolving in their lipid plasma membrane. In addition to concentration differences, other factors can affect the diffusion of a drug across the BBB such as lipophilicity and molecular weight. Only lipid-soluble small molecules with a molecular weight of 400 Daltons can cross the BBB. Facilitated diffusion is a form of carrier-mediated endocytosis in which solute molecules bind to specific membrane protein carriers that trigger a conformational change in the protein. This results in a carrying of the substance to the other side of the membrane, from high to low concentration (passive diffusion). This mechanism contributes to the transport of various substances including amino acids, nucleoside, small peptide, monocarboxylates, and glutathione. For almost all other substances, including essential materials such as glucose and amino acids, transport proteins (carriers), specific receptor-mediated or vesicular mechanisms (adsorptive transcytosis) are required to pass the BBB.

Carrier-mediated transport (CMT) or carrier-mediated influx processes involve putative proteins that facilitate the movement of poorly permeable solutes across cellular membranes. CMT systems can be exploited for brain drug delivery after reformulating the drug in such a way that the drug assumes a molecular structure mimicking that of the endogenous ligand. Gabapentin (a γ-amino acid) successfully crosses the BBB because the structure does mimic that of an α-amino acid and is recognized by large neutral amino acid transporter [87]. The uptake of nutrients from blood into the brain is facilitated by the solute carrier (SLC) transporter families. These influx carriers are involved in the transport of a broad range of substrates including glucose, amino acids, nucleosides, fatty acids, minerals and vitamins in various human tissues, including the brain.

The active efflux transport is responsible for extruding drugs from the brain, and this mechanism is a major obstacle for the accumulation of a wide range of biologically active molecules in the brain. The ATP-binding cassette (ABC) transporter P-glycoprotein and multidrug-resistant protein (MRP) represent the principle efflux mechanism of these agents [88]. The most abundantly present component of this system is efflux P-glycoprotein, which is a product of the *ABCB1* gene. Inhibition of P-glycoprotein in preclinical studies has enhanced the penetration of paclitaxel into the brain, indicating the feasibility of achieving improved drug delivery to the brain by suppression of P-glycoprotein [89].

Endocytosis and transcytosis allow the internalization, sorting, and trafficking of many macromolecules. Endocytosis is a process where molecules from the circulation are internalized in vesicles and are directed to endosomes or lysosomes within the cell. Endocytosis can be isolated into bulk-phase (fluid phase or pinocytosis) endocytosis and mediated endocytosis (receptor and absorptive mediated). Bulk-phase endocytosis is the noncompetitive, non-saturable, temperature, and energy dependent non-specific uptake of extracellular fluids. Transcytosis refers to the transcellular movement of molecules.

Receptor-mediated transcytosis across the BBB has been explored more actively because of its high specificity. Receptor-mediated transport is mainly employed in the transport of macromolecules like peptides and proteins across the BBB, by conjugating the substance with various ligands. It is an important transport mechanism of predominant interest in drug delivery. Large molecules which are necessary for the normal function of the brain are delivered to the brain by specific receptors. These receptors are highly expressed on the endothelial cells forming the BBB. Therapeutic compounds can cross the BBB after association/conjugation to these specific ligands. Receptor-mediated transcytosis has been demonstrated for insulin, insulin-like growth factors (IGF-1 and IGF-2), leptin, the low-density lipoprotein receptor-related protein (LRP) [90].

The insulin receptor (IR) is a large protein with a molecular weight of 300 kDa. A novel study reports that a genetically engineered human/mouse chimeric form of the human insulin receptor monoclonal antibody (HIRMAb), in an adult Rhesus monkey, has been rapidly transported to the inner primate brain after intravenous administration, suggesting its potential for delivering drugs across the BBB [91].

Low-density lipoprotein receptor related proteins 1 and 2 (LRP1 and LRP2) are multifunctional, multi-ligand scavenger and signaling receptors. They can interact with a diverse range of molecules and mediators including ApoE, tissue plasminogen activator (tPA), plasminogen activator inhibitor 1 (PAI-1), lactoferrin, melanotransferrin, α2 macroglobulin (α2 M), receptor-associated protein (RAP), HIV-1 TAT protein, heparin cofactor II, heat shock protein 96 (HSP-96) and engineered angiopeps. Another group of LRP ligands, known as angiopeps, has also been reported to be highly effective BBB targeting ligand. The most studied is angiopep 2 which has shown greater transcytosis capacity and parenchymal accumulation.

Adsorptive transcytosis facilitates the transport of large peptides such as IgG, histone, albumin, native ferritin, horse radish peroxidase and dextran. Adsorptive-mediated transcytosis relies on the interaction of a ligand with moieties expressed at the luminal surface of cerebral endothelial cells. Several peptides allow the intracellular delivery of polar, biologically active compounds *in vitro* and *in vivo* [92]. These peptides have been successfully used as vectors for delivery of drugs that are P-gp substrates by effectively bypassing the P-gp in the BBB. However, because it is a non-specific process, the adsorptive process also occurs in the blood vessels and in other organs. This poses a challenge for both achieving therapeutic concentration in the brain and limiting the drug distribution in non-target organ.

Cell penetrating peptides (CPPs) and cationic proteins (e.g., albumin) are commonly used to enhance brain drug delivery via adsorptive-mediated transcytosis. A large variety of cargo molecules/materials have been effectively delivered into cells via CPPs, including small molecules, proteins, peptides, fragments of DNA, liposomes and nanoparticles. The transcription factor Tat, involved in the replication cycle of human immunodeficiency virus (HIV), was demonstrated to penetrate into cells [93]. SynB vectors are a new family of vectors derived from the antimicrobial peptide protegrin 1 (PG-1). These peptides are able to interact with the cell surface and cross the plasmatic membrane. Furthermore, the internalization of these peptide vectors does not depend on a chiral receptor, since the d-enantio form penetrates as efficiently as the parent peptide (l-form), and retro-inverso sequences exhibit identical penetrating activity. Adenot and colleagues studied brain uptake of a number of free and SynB3 vectorized chemotherapeutic agents using both *in situ* brain perfusion and *in vitro* BBB/cell model [94]. They reported that SynB3’s conjugation with various poorly brain-penetrating drugs enhanced their brain penetration with no effect on tight junction integrity.

## 4. Therapeutic Approaches in Brain Metastases

Important results have been obtained in the recent past in the management of patients affected by BM using surgery, radiation therapy, and both surgery and radiotherapy. The most interesting data was demonstrated in patients with single BM in whom surgical resection followed by whole-brain cranial irradiation resulted in a median survival of 40 weeks, compared with 15 weeks in those treated with radiation alone. Currently, the treatment of BM requires a multidisciplinary approach tailored to each individual patient. The treatment algorithm for BM changes depending on factors such as primary histology and other clinical characteristics of patients, as well as available therapeutics options in each clinic. Many patients are treated with a combination of treatments, and decisions must take into account factors such as patient’s age, functional status, primary tumor type, extent of extracranial disease, prior therapies, and number of intracranial lesions.

### 4.1. Surgery

The surgical decision is first made depending on the patient’s clinical situation. Surgery is often used in patients with recursive partitioning analysis class I/II, a single metastasis, and a minimal or controlled systemic tumor. Furthermore, candidates for surgery should have a life expectancy of at least six months. Only surgical resection allows the rapid debulking of a large, immediately life-threatening tumor, making it beneficial to patients with neurological signs and symptoms related to metastatic disease. Surgery relieves mass effect and symptomatic intracranial hypertension, restores CSF flow, and lower steroid dependence through a reduction in peritumoral edema. Medically uncontrollable seizures due to BM are an indication for surgical resection. Surgical resection permits, moreover, the provision or confirmation of a correct pathological diagnosis. Patients who have large cystic lesions in the eloquent area with poor performance status may undergo palliative insertion of an Ommaya reservoir for cystic tumor management. Furthermore, patients diagnosed with leptomeningeal metastases may benefit from insertion of Ommaya reservoir and intrathecal/intraventricular drug delivery. The role of surgery in the management of multiple BM is still controversial. Traditionally, the identification of multiple BM was considered a contraindication to surgical intervention, and most patients with multiple BM were treated exclusively with WBRT. In a retrospective study, Bindal *et al.* compared patients with multiple BM who underwent complete surgical resection with those who underwent partial removal, and found that patients undergoing excision of multiple or single BM had significantly longer survival than patients who underwent partial tumor resection [94]. In another study, the authors demonstrated that, in treated patients with multiple BM, significant variables for shorter survival were an age greater than 60 years, a Karnofsky Performance Score (KPS) of less than 70, an incomplete surgical removal, and the presence of extensive systemic cancer. The presence of multiple lesions was not a significant predictor of shorter survival, suggesting that resection of multiple BM is a worthwhile approach [95]. However, the most important factor to consider when selecting patients for resection of multiple BM is the extent of the systemic cancer (both the primary tumor and the non-cerebral metastases) [96].

### 4.2. Radiotherapy

The goal of WBRT includes treatment of the known metastases and prevention of future ones. Median survival after WBRT alone is 3–6 months. This relatively short survival does not necessarily reflect a failure of WBRT, since more than half the deaths are related to progressive systemic disease rather than BM. Radiotherapy seems to be related only to a prolonged progression-free survival, with better control of seizures, but with no substantial differences in overall survival. However, WBRT is associated with late brain toxicities, which range in severity from mild deficits in cognitive dysfunction to overt dementia in up to 11% of patients depending on the population studied, the length of follow-up, and the type of chemotherapy employed [97–99]. Patients treated with radiotherapy are at high risk of developing some complications such as post-radiation leukoencephalopathy, characterized by dementia, gait disturbance, incontinence, and a deficit in attention and executive functions. There is consensus among radiation oncologists to apply a relatively short course of radiation therapy with 30 Gy total dose given in 300 cGy fractionated dose, five times per week. In special palliative settings a more accelerated course with 400 cGy fractionated dose up to 20 Gy total dose can be considered. Based on the available class I and class II evidence, surgical resection followed by WBRT is an effective treatment for patients with single, surgically accessible, BM who have controlled extracranial disease and are in good general condition [100]. In multiple BM, WBRT permits the control, in 70%–90% of cases, of presenting neurological symptoms, without causing acute neurological side effects [101]. Prophylactic cranial irradiation (PCI) in patients with small cell lung cancer (SCLC) has been investigated as a strategy to prevent dissemination to the brain. PCI resulted in a reduction in the incidence of BM from 18% to 8%, but did not impact overall survival [102]. Importantly, PCI results in lower rates of both immediate and delayed recall, suggesting that the use of PCI impairs memory function in treated patients [102].

### 4.3. Radiosurgery

The term radiosurgery refers to a technique of irradiation that allows for the concentration of a high dose of ionizing radiation on a target volume with a high geometric precision. SRS uses a linear accelerator (LINAC) or multiple cobalt-60 sources (gamma-knife) to targets of 3–3.5 cm maximum diameter. The local tumor-control rate was high after SRS, with an 85% control rate at 1 year, and a 65% control rate at 2 years [103]. SRS is particularly useful for patients unable to tolerate surgery and for patients with lesions inaccessible to surgery. SRS is reported effective in the treatment of BM that tends to be resistant to conventional radiation therapy, such as melanoma and renal cell carcinoma. Prognostic factors in patients receiving SRS were the KPS score, total intracranial volume and the presence of active systemic disease.

The role of WBRT in patients treated with SRS is still controversial, especially for patients with relatively radioresistant tumors. While recent data established that the addition of WBRT to SRS significantly improves local tumor control, an overall survival benefit has not been demonstrated. Patients report that the addition of WBRT causes more memory impairment, depression, poor concentration, and hair loss than SRS alone. Andrews *et al*. [5] published the first randomized trial comparing SRS combined with WBRT to WBRT alone (RTOG 95-08). For patients with a single unresectable metastasis, SRS was found by intention-to-treat analysis to confer a significant survival benefit. Additionally, the SRS group showed a significant improvement in KPS and decreased steroid use at six months. There was no significant survival benefit for patients with multiple metastases. Cyberknife (CK) is a new radiotherapy method that can give higher therapeutic doses directly to the tumor. CK show the natural technical advantage in fixation, real-time authentication and dynamic tracing. Chang *et al.* using CK, radiated single-fractionated doses of 10–36 Gy in 84 BM in 72 patients, obtained a tumor control rate of 95% and a radiation damage rate of 4% [104]. In a recent review, 40 patients affected by brain metastases were treated with CK. One week after CK treatment, symptomatic improvement occurred in 90.0% of patients, with a 77.8% local control rate at three months, a therapeutic effective rate of 94.1%, and a one-year survival rate of 67.5% [105].

### 4.4. Chemotherapy

Chemotherapy has traditionally played a limited role in the treatment of BM and has been reserved for patients who have failed other treatment modalities. Although BBB is interrupted, brain therapeutic levels of many drugs do not remain long enough or at high enough concentrations to ensure cell apoptosis. Furthermore, the drug distribution is not uniform, with a preferential concentration in the necrotic area and a rapid diffusion into normal brain. Treatment efficacy is determined by the sensitivity of tumor cells to chemotherapeutic agents. BM from NSCLC and breast cancer are less sensitive to chemotherapy. There is, therefore, a strict relation between metastatic and primary tumor chemosensitivity and the choice of chemotherapeutic regimen. In patients with BM from breast cancer, cisplatin and etoposide yielded a high objective response rate of 55% in CNS [106]. Recently, a new class of chemotherapeutic agents that own the ability to cross the physiological BBB holds results for patients affected by BM.

Topotecan is a semi-synthetic camptothecin derivative that selectively inhibits topoisomerase I in the S-phase of the cell cycle, interfering with the replication and transcription processes in the tumor cell, which eventually leads to cell death. In addition to its well-established activity against primary tumors, topotecan freely penetrates the BBB and measurable levels of topotecan and its metabolites can be detected in the cerebro-spinal fluid. Topotecan monotherapy was evaluated in 20 SCLC patients with asymptomatic BM after failure of first-line chemotherapy, but without radiation therapy, suggesting that topotecan can induce a high response rate in SCLC BM [107]. Wong and Berkenblit affirm that topotecan, especially in patients with SCLC or breast cancer, has shown excellent response rates against BM and may effectively combine with WBRT and other chemotherapeutic drugs [108]. A recent study confirms the good efficacy of topotecan in SCLC but, in contrast with other studies, do not support the use of topotecan in brain metastases arising from other tumors. In addition, the ability of topotecan to cross the BBB suggests that it may also have a prophylactic role against brain metastases from SCLC [109].

Temozolomide (TMZ) is a novel oral alkylating agent that has demonstrated efficacy in the treatment of a variety of solid tumors. Because of its small molecular weight, TMZ crosses the BBB, and, in addition, can be administered orally. TMZ is also associated with a low incidence of severe adverse events. Based on the clinical pharmacology of TMZ, it has been suggested that TMZ may be effective in the prevention and treatment of brain metastases. Paul *et al*. [110] have reported that, among 40 patients with advanced melanoma treated with TMZ, the incidence of CNS relapse was lower in patients treated with TMZ. Only two patients of 19 (10%) treated with TMZ developed CNS metastasis.

## 5. Molecular Targeted Therapy

Elevated expression or mutation of receptors and intracellular downstream effectors has been demonstrated in metastatic progression. These pathways are controlled by the binding of growth factors to tyrosine kinase receptors. Specific targeting of these signaling pathways that lead to altered cellular proliferation and cell migration and invasion could provide new targets for BM treatment. Targeted therapies block activation of oncogenic pathways, either at the ligand–receptor interaction level or by inhibiting downstream signal transduction pathways, thereby inhibiting growth and progression of disease (Table 2). Because of their specificity, targeted therapies should theoretically have better efficacy and safety profiles than systemic cytotoxic chemotherapy or radiotherapy.

### 5.1. Trastuzumab

The incidence of BM is particularly high in patients with human epidermal growth factor receptor 2 (HER2) positive breast cancer [111]. With the exception of HER2 that does not have a ligand-binding domain, the rest of the HER family receptors, upon ligand binding to their extracellular domain, forms either homodimers or heterodimers that initiate their intrinsic tyrosine kinase activity controlling the activation of various downstream effectors pathways. Intracardiac injection of the control and HER2-transfected 231BR cells produced similar numbers of brain micrometastases, but the HER2 transfectants produced 2.5- to 3-fold greater numbers of large BM [112]. These data provide the evidence that HER2 overexpression changes the natural history of breast cancer to promote outgrowth of tumor cells in the brain [112].

Trastuzumab is a humanized monoclonal antibody that targets the extracellular domain of HER2. It has been approved for the treatment of metastatic breast cancer, alone or in combination with chemotherapy, for patients with tumors that overexpress the HER2 receptor. In a retrospective study with patients affected by HER2-positive breast cancer that developed BM, Nam and colleagues reported a median OS of 13 months in patients who received trastuzumab compared with 4 months in those who did not receive trastuzumab and 3 months in patients with HER2-negative tumors [113]. Bartsch and colleagues also evaluated the effect of the continuation of trastuzumab after diagnosis of BM for 17 patients, in comparison with a cohort of 36 patients with HER2 overexpressing tumors not treated with trastuzumab after WBRT [114]. In this study, KPS and trastuzumab were associated with better overall survival, with a trend towards longer time to in brain progression. The results demonstrated that trastuzumab may act synergistically with radiation in a HER2 level-dependent manner encouraging further assessment in combination with WBRT [114]. However, a metanalysis by Bria and colleagues used the data from 3 large phase III trials in the adjuvant setting, the National Surgical Adjuvant Breast and Bowel Project (NSABP), the Herceptin Adjuvant trial (HERA), and the NCCTG N9831, indicating that the incidence of CNS disease was significantly higher in the trastuzumab-treated patients when compared to the non-trastuzamab-treated patients [115]. To evaluate the potential of effects on the CNS, a four-week toxicology study with weekly intrathecal administration of trastuzumab was performed in cynomolgus monkeys at doses of 0, 3, or 15 mg. No trastuzumab-related effects on body weight, clinical signs, neurological function, clinical pathology, or anatomic pathology were noted. The applied doses and CSF concentrations achieved in the current study exceeded those reported in patients after intrathecal administration. The results support future studies for intrathecal application of trastuzumab in patients with brain metastases in HER2-positive breast cancer [116]. Recent studies have examined the influence of patient characteristics on survival and have attempted to identify subgroups of patients with substantially different outcomes in order to tailor therapy and to rationalize the design, stratification and interpretation of clinical trials. The Radiation Therapy Oncology Group (RTOG) recursive partitioning analysis (RPA) classification based on clinical factors (KPS, age, and control of extracerebral disease) the prognostic indicator for patients with BM [117].

### 5.2. Lapatinib

Lapatinib is a small-molecule tyrosine kinase inhibitor that inhibits the EGFR and HER2 and has the potential to be used when trastuzumab resistance develops. In mice with BM, treatment with lapatinib results in a statistically significant decrease in phosphorylated HER2, suggesting that pharmacologically relevant levels are achieved in CNS metastatic lesions [118]. Interestingly, lapatinib can cross the BBB and has modest activity in breast cancer metastases in the CNS [119]. A recent study demonstrated that lapatinib monotherapy 750 mg given twice daily can exert some efficacy and has potential as a clinically meaningful treatment option for Japanese HER2-overexpressing breast cancer patients with BM after cranial radiation [120]. *In vitro*, lapatinib inhibited the phosphorylation of EGFR, HER2, and downstream-signaling proteins, cell proliferation, and migration in 231-BR cells. Mice bearing HER2 overexpressing xenographs which received lapatinib developed less frequent BM compared to control [120].

Several trials have demonstrated the safety and efficacy of lapatinib alone and in combination with capecitabine, paclitaxel or endocrine therapy in patients with advanced HER2-positive breast cancer [119]. In the EGF105084 study, 242 patients with HER2 overexpressing BC with CNS disease received lapatinib after cranial radiotherapy [121]. Clinically significant CNS objective responses were observed in 20% of patients treated with lapatinib plus capecitabine after disease progression on single-agent lapatinib. Overall, 40% of patients achieved a 20% or greater reduction in the volume of CNS lesions. Lapatinib seems to be associated with regressions of BM in patients who have progressed despite trastuzumab and radiotherapy [121].

### 5.3. Erlotinib and Gefitinib

Erlotinib and gefitinib are small molecules, reversible inhibitors of the tyrosine kinase domain of the EGFR resulting in the loss of autophosphorylation and subsequent downstream signaling through the RAS-RAF-MEK pathway. These small molecules have demonstrated efficacy in patients with relapsed NSCLC and as initial therapy for patients with advanced NSCLC and sensitizing EGFR mutations. Specific activating mutations within the tyrosine kinase domain of EGFR have been identified. The missense mutation L858R in exon 21 and the in-frame deletion in exon 19, nested around the amino acid residues 747 to 750 of the EGFR polypeptide, account for >85% of all clinically important mutations related to tyrosine kinase inhibitors (TKI) sensitivity. The detection of EGFR mutations in tumor tissues has been applied for predicting the response of TKI treatment and hence guiding the treatment for advanced NSCLC. However, brain tissue is the only available tissue for the determination of EGFR status, and the question of concordance between primary and metastatic EGFR status becomes crucial for therapy [122]. Studies on paired brain metastases/NSCLC suggest a possible discordance, but they are too few and are essentially insufficient to clarify this problem. A panel of 30 EGFR kinase domain mutations that were recently reported in NSCLC patients was cloned and expressed for analysis of kinase activity, transforming potential, and drug sensitivity. Most somatic mutations of EGFR are associated with 60%–80% response rates in patients treated with gefitinib or erlotinib [123]. Some initial case reports have showed activity of gefitinib and erlotinib on BM from NSCLC, suggesting a potential role of TKI in the treatment of NSCLC patients with metastatic CNS disease.

Ishida *et al.* administered gefinitib, in two women, affected by differentiated adenocarcinoma of the lung with BM without any previous systemic therapy. In both patients, the metastatic brain lesions reduced notably after gefinitib treatment [124]. Similarly, in two patients that received gefitinib orally, the disappearance or the reduction of the BM has been demonstrated [125]. Tang *et al.* reported the case of a woman with diffuse BM from lung cancer who experienced total regression of the metastases under gefitinib treatment. The tumor was positive for an EGFR exon 19 deletion mutation. She was treated with gefitinib 250 mg/day. One year later, the diffuse brain metastases had totally resolved [126].

Recently, a case of NSCLC with CNS metastases harboring a rare EGFR double-activating mutation has been reported as showing a good clinical response to erlotinib [127]. In another case, a complete remission in brain disease from NSCLC using erlotinib was obtained [128]. In this case, the presence of somatic mutation in EGFR gene has been associated with a higher responsiveness to erlotinib. The authors sequenced exons 18–21 of the EGFR gene using DNA extracted from tumor and normal lung tissue obtained at a previous resection: the L858R mutation was detected. This point mutation in the activation loop of the kinase domain is linked to erlotinib responses [128]. In a phase II trial, Ceresoli and colleagues assessed the efficacy of gefitinib in 41 patients with NSCLC metastatic to the brain. In total, disease control rate was observed in 11 patients. The authors suggested that gefitinib, at the standard dose of 250 mg/day, can be active on brain disease in NSCLC patients [129]. In a second trial, gefitinib or erlotinib was given in chemotherapy and RT-naïve patients with adenocarcinoma of the lung and asymptomatic BM [130]. The disease control rate was 82.6%; 47.8% of patients had to receive WBRT during the course of their disease for symptom control. Although patients who received WBRT post-study had longer survival rates when compared to those who did not, this was not statistically significant. In a recent study, the risk of CNS progression in patients with stage IIIB/IV or relapsed NSCLC with somatic EGFR mutations who were treated with gefitinib or erlotinib as their initial therapy for advanced NSCLC has been evaluated. The data demonstrated that the incidence of CNS progression in patients with advanced EGFR-mutated NSCLC initially treated with gefitinib or erlotinib was 28% after a median potential follow-up of 42.2 months. The authors evidenced a lower risk of CNS progression in patients with somatic EGFR mutations initially treated with gefitinib or erlotinib for advanced NSCLC compared with published rates of CNS failure in NSCLC patients treated with systemic chemotherapy plus local therapy for locally advanced disease. In addition, the development of CNS metastases was a relatively late event in our patients, occurring at a median of 19 months following the initiation of gefitinib or erlotinib [131].

It was observed that the characteristics of patients with predominant EGFR mutations associate strikingly with those of gefitinib responders. These results suggest that EGFR mutations may predict the responsiveness of NSCLC to gefitinib. However, the data in this limited study did not provide a statistically significant result, but did exhibit the possibility that a similar relationship between EGFR mutations and the efficacy of gefitinib exists in BM from NSCLC [132]. In a novel study, patients treated with gefitinib with concomitant WBRT had superior time to progression (TTP) of BM compared with gefitinib alone group. The data, showed, also, a significant survival benefit of gefitinib with concomitant WBRT compared with gefitinib alone (23.4 *vs.* 14.83 months). The authors hypothesized that the long duration of disease control of CNS lesions contributed to the survival advantage. In summary, gefitinib and concomitant WBRT showed an advantage over gefitinib alone in terms of PFS, OS and TTP of CNS lesions [133].

### 5.4. Multitarget Tyrosine Kinase Inhibitors

The MAPK pathway is a major intracellular signal transduction pathway that is responsible for cellular proliferation, gene expression, differentiation, mitosis, cell survival, and apoptosis.

Sorafenib is an orally active multikinase inhibitor that blocks intracellular kinases in the Raf/MEK/ERK pathway that are involved in tumor proliferation, such as Raf-1, as well as those promoting angiogenesis, including VEGFR-2, VEGFR-3, FLT, PDGFR-*b*, FMA, RET, and c-KIT. In phase III Treatment Approaches in Renal Cancer Global Evaluation Trial (TARGET), sorafenib treatment was associated with a twofold increase in median PFS compared with placebo [134]. A recent study that reported five cases of intracerebral hemorrhage in metastatic renal cell carcinoma (RCC) patients with BM following treatment with either sorafenib or sunitinib raised the concern that antiangiogenic therapies, in patients with BM, may increase the risk for CNS hemorrhage [135]. However, Massard and colleagues reported that patients that received sorafenib were less likely to develop BM when compared to the control group (3% *vs.* 12%, respectively) [136]. Sorafenib may reduce metastases by suppressing the progression of visceral disease or by inhibiting brain metastasis angiogenesis. In a recent study, a patient affected by renal cell carcinoma with multiple BM was successfully treated with multimodal therapy including sorafenib [137]. Sorafenib has also shown promise in the treatment of patients with advanced or metastatic thyroid carcinoma. In a patient affected by follicular thyroid carcinoma and BM, symptoms and signs improved dramatically and continuously after initiation of sorafenib treatment [138].

Activating mutations in the serine/threonine kinases BRAF, and NRAS were identified in 66% and 15% [139] of melanoma cell lines, respectively, establishing MAPK signaling as a new therapeutic target in melanoma. Over 75% of BRAF mutations are characterized by the substitution of valine by glutamic acid at residue 600 (V600E) [140,141]. A phase I/II trial in which sorafenib was given in combination with carboplatin and paclitaxel reported a high response rate and longer PFS than with standard chemotherapy in metastatic melanoma patients [142].

The most promising results in patients with BRAF^mut^ melanoma have been seen with drugs designed to selectively target the mutated and activated form of the BRAF kinase. The three drugs in clinical use or undergoing investigation in human clinical trials are LGX818, vemurafenib, and dabrafenib. These inhibitors are associated with specific toxicities and with the rapid development of resistance.

LGX818 is a type 1 BRAF inhibitor under investigation in phase I clinical trials, both as a single agent (NCT01436656) and in combination with other targeted therapies (NCT01543698) [143].

Vemurafenib is a potent inhibitor of mutated BRAF. It has marked antitumor effects against melanoma cell lines with the BRAF^V600E^, mutation but not against cells with wild-type BRAF [144,145]. When used as a first-line agent in BRAF^V600E^ metastatic melanoma, vemurafenib had a Response Evaluation Criteria in Solid Tumors (RECIST) response rate 63 of 53%, a median PFS of 6.9 months, and a median OS of 13.6 months, compared with conventional dacarbazine chemotherapy’s response rate of 8%, median PFS of 1.6 months, and median OS of 10 months [146]. A phase I study in patients with BM showed intracranial activity, [147] and a phase II study is currently underway (NCT01378975).

Dabrafenib is a reversible and potent adenosine triphosphate-competitive inhibitor that selectively inhibits the BRAF^V600E^ kinase. The phase I study showed dabrafenib to be safe and tolerable, to demonstrate activity in BRAF^V600E^ and BRAF^V600K^ melanoma, and to be the first drug to show activity in melanoma metastases in the brain [143]. The results of phase II BM trial (BREAK-MB)11 [148,149] suggest that dabrafenib may be an effective adjunct for the treatment of BM, and that it warrants consideration as first-line therapy in patients with brain metastases, and with advanced extracranial disease. Additionally, dabrafenib was well tolerated, with the exception of intracranial hemorrhage, which occurred in 6% of patients [143]. Dabrafenib was also evaluated in a multicenter, open-label, phase 2 trial, in patients with Val600Glu or Val600Lys BRAF-mutant melanoma metastatic to the brain. The results of this study show that dabrafenib is well tolerated and can represent a valid therapeutic strategy in patients with BRAF-mutant melanoma with BM either previously treated or not [150].

Sunitinib is a new, orally administered, small molecule that inhibits members of the split-kinase domain family of receptor tyrosine kinases (RTKs), including the VEGFRs types 1 and 2, PDGFR-α and PDGFR-β, the stem cell factor receptor c-KIT, the FLT3 and RET kinases. Sunitinib exhibits potent antiangiogenic and antitumor activity. Valid clinical activity has been observed in metastatic renal cell carcinomas and imatinib-resistant gastrointestinal stromal tumors, leading to regulatory approval in these two indications. Sunitinib or its metabolite penetrated the CNS of monkeys with rapid clearance, but does not appear to accumulate. In an open-label expanded study for the use of sunitinib in metastatic RCC, which included patients with BM, a positive response in intracranial disease was observed. In addition, 52% of patients had stable disease for at least three months [151]. In a phase II study, antitumor activity and safety of sunitinib in patients with pretreated NSCLC and irradiated BM were evaluated [152]. Patients received sunitinib 37.5 mg on a continuous daily dosing schedule. Median progression-free survival was 9.4 weeks, and median overall survival was 25.1 weeks. Serious neurologic adverse events occurred in six patients (9%), and none were treatment-related. No cases of intracranial hemorrhage were reported [152].

### 5.5. Cediranib

Cediranib (AZD2171) is a potent oral, pan-VEGF receptor tyrosine kinase inhibitor with activity against PDGF receptors and c-Kit. In an experimental study, BM-selected variant cells were recovered after three cycles of injection into the internal carotid artery of nude mice and harvest of BM, resulting in variants termed MDA-231 BR1, -BR2 and -BR3. Brain metastatic lesions of the selected variants contained significantly more CD31-positive blood vessels than metastases of the non-selected cell line. The variants selected from BM released significantly more VEGF-A and IL-8 into culture supernatants than the original cell line, and more VEGF-A RNA when cultured in normoxic conditions. Mice injected with MDA-231 BR3 into the carotid artery were treated with the VEGF-receptor tyrosine kinase inhibitor PTK787/Z 222584. Oral administration of the inhibitor resulted in a significant decrease in brain tumor burden, reduced CD31-positive vessels in the brain lesions and incidence of PCNA positive tumor cells, and increased apoptosis in the tumor [31]. In a phase II study, it has been demonstrated that AZD2171 induced vascular normalization and reduction of vasogenic brain edema in recurrent glioblastomas [153]. In an experimental study, an hematogeneously-disseminated model of BM derived from a human androgen-independent prostate cancer, was used [154]. BM in the DU145/RasB1 model occur as large, expansive rounded lesions with marked peritumoral edema, as well as small infiltrative lesions. AZD2171 treatment resulted in a decreased blood volume within the center of the large tumors. Histological sections confirmed central necrosis of large tumors and that the blood vessels at the rim of the AZD2171-treated tumor were still dilated with hypertrophic endothelial cells [154]. Similarly, with a model of advanced prostate cancer metastatic to skeleton and brain, it has been demonstrated that antiangiogenic treatment inhibited the growth of metastases in bone and brain, and reduced the morbidity and mortality of tumor-bearing mice [155].

### 5.6. Bevacizumab

Bevacizumab is a monoclonal antibody that binds VEGF-b, inhibiting angiogenesis. Inhibition of VEGF by bevacizumab will not only affect endothelial cells but also the tumor vasculature, suppressing new blood vessel growth and the existing vasculature. Concerns about the risk of intracranial hemorrhage initially precluded use in patients with brain metastases. A phase II trial of bevacizumab for NSCLC reported intracranial hemorrhage in patients developing cerebral metastasis during treatment, although the incidence was <1% [156]. However, successive studies with patients with various primary cancers demonstrated no significant increase in the risk of intracranial hemorrhage in patients with BM treated with bevacizumab [157]. Findings from a multicenter prospective phase II trial showed that the addition of bevacizumab to various chemotherapy agents in patients with NSCLC and BM is safe, even though a low incidence of CNS hemorrhage was reported [158]. De Braganca *et al*. suggests that bevacizumab administered with therapeutic intent for treatment of active CNS metastases may be effective safe and effective, especially for small lesions that are less likely to hemorrhage [159]. In a new study, five patients with BM received bevacizumab combined with paclitaxel. The majority of adverse events were mild to moderate in intensity. Hypertension and proteinuria were common, and neuropathy was controlled with modification of the paclitaxel dose [160].

### 5.7. Other Molecules

mTOR inhibitor rapamycin and its analogs are lipophilic, demonstrate BBB penetration, and have shown promising antitumor effects in several types of refractory tumors. The effects of different dose of mTOR inhibitors (rapamycin, Temsirolimus-CCI-779) on cell invasion in two brain metastatic breast cancer cell lines (MDA-MB231-BR and CN34-BrM2) were examined [161]. The two mTOR inhibitors, rapamycin and CCI-779, inhibited the invasion of brain metastatic cells only at a moderate concentration level, which was lost at higher concentrations secondary to activation of the MAPK signaling pathway. *In vivo*, a significant decrease was noted in the average number of micro and large metastatic lesions as well as the whole brain GFP expression in the CCI-779. Combined with the brain MEK inhibitor SL327, high-dose CCI-779 significantly reduces the BM, and the combination treatment prohibited perivascular invasion of tumor cells and inhibits tumor angiogenesis *in vivo* [161].

In a patient with multiple brain lesions from non-small cell lung cancer, a weekly dose of 250 mg/m cetuximab was administered for 3 months. The target lesion showed enhancement of radiolabeled cetuximab on scintigraphy, demonstrating an accumulation of cetuximab in BM [162].

Enzastaurin is a protein kinase C inhibitor with antitumor activity. This study was designed to determine if maintenance enzastaurin improved the outcome of WBRT in lung cancer patients with BM. Enzastaurin was well tolerated but did not improve overall survival or progression-free survival after WBRT in patients with BM [163].

## 6. Conclusions

BM currently represents an important cause of cancer morbidity and mortality. Although many patients with BM die as a result of extracranial disease progression, it is important to note that a significant amount suffer from the local tumor progression in the CNS. The main prognostic factors for BM patients are age, performance status, control of primary tumor, absence of extracranial disease, and number of brain lesions. Surgery, stereotactic radiotherapy, WBRT and systemic methods have been integrated in the therapeutic options, depending on the number, site and size of secondary brain lesions. Delivering concomitant chemotherapy may improve tumor local control but does not improve overall survival and is thus not recommended for the routine treatment of BM patients.

Class I patients with good performance status and limited extracranial disease surgical resection followed by post-operative WBRT represent, in terms of improving tumor control at the original site of the metastasis and in the brain overall, an optimal choice. Class II evidence suggests that larger lesions (>3 cm) or those causing significant mass effect (>1 cm midline shift) may have better outcomes with surgical resection, whereas radiosurgery may offer slightly better local control rates for radioresistant lesions (*i.e.*, melanoma, renal cell, *etc.*). One notable treatment combination in need of further study involves the concept of applying SRS to the surgical resection cavity post-operatively instead of post-operative WBRT. No evident prospective data yet exists to support a few retrospective case series, suggesting that both local control rates and even survival are enhanced by this post-operative SRS option.

Tumors are biologically heterogeneous and contain subpopulations of cells with different angiogenic, invasive, and metastatic properties. Additionally, some primary and secondary tumors may have unique biological signatures that may respond to targeted agents and biological modulation. Histology-specific brain metastasis trials may also help answer important therapeutic questions regarding radioresistant lesions *versus* other common histologies. Most studies thus far have not specifically addressed differences in histological subtype despite the fact that management of extracranial malignancies differs widely based on cancer histology. Novel researchers show that to produce brain metastasis, tumor cells must reach the vasculature of the brain, attach to the microvessel endothelial cells, extravasate into the parenchyma, proliferate, and induce angiogenesis. Antiangiogenic therapy has been demonstrated to represent a promising novel approach to the treatment of BM. ECM, proteases, cell adhesion molecules and their related signaling pathways show an important role in BM invasion and could be selectively attacked to inhibit the metastatic invasion. Different antiangiogenic strategies have been developed: inhibition of proangiogenic factors and/or receptors and/or downstream cell signaling; inactivation of ECs; and inhibition of cellular adhesion molecules and/or ECM remodeling. The most promising include antiangiogenic drugs, inhibitors of v-RAF murine sarcoma viral oncogene homolog B1 (BRAF) for BRAF ^V600E^ mutated melanoma, and inhibitors of the epithelial growth factor receptor for non-small cell lung cancer [122,143,146,150]. Several clinical trials of antiangiogenic therapies are being conducted, but investigators are still concerned about how to achieve the maximum benefit from them and how to monitor patient response. However, no clear benefits for patients have been reported regarding the use of single antiangiogenic drugs. Considering the multitude of molecular entities and signaling pathways regulating the proliferation and cellular survival/cell death, the inhibition of a singular target could not be sufficient to suppress neoplastic progression [83]. Furthermore the targeting of one molecule or pathway may lead to the increased activity of other pathways, which may then sustain angiogenesis. However, there are several factors underlying the disappointing results in BM treatment, including limited tumor cell drug uptake, intracellular drug metabolism, inherent tumor sensitivity to chemotherapy and cellular mechanisms of resistance, risk of intracranial bleeding, and the desire to include only patients with more than modest life expectancy. Understanding the genetic bases of BM and of the invasive behavior, in particular the differentiated gene expression in distinct areas of the same tumor during progression, may suggest new molecular targets to overcome the mechanisms of multidrug-resistance of the actual therapeutic approaches and to simultaneously attack different crucial biological events of the metastatic process.

In this complex field, improving specific selective drug-delivery systems to lead the diffusion of drugs, engineered monoclonal antibodies, and other therapeutic molecules into the CNS by overcoming BBB is crucial. Actually, the brain represents a preferential site of metastasis because many of the new therapies cannot sufficiently cross the BBB. In this way, nanotechnology provides a unique opportunity to combat cancer on the molecular scale through careful engineering of nanomedicines to specifically interact with neoplastic cells and inhibit their function. It is also possible to take into neoplastic tissue, novel selective contrast enhancement molecules to visualize brain tumors and to study *in vivo* all of their characteristics, such as cellular proliferation, angiogenesis, necrosis, tumor-safe tissue interface, and edema [164,165]. There are significant opportunities to investigate the use of nuclear imaging techniques, such as positron emission tomography (PET) and single-photon emission computed tomography (SPECT), for development of radiolabeled nanoparticles targeting cancer. The surface of nanoparticles can be modified to achieve targeted delivery and improved biocompatibility, and various compounds may be encapsulated inside for multiple functions. Nanoparticle-based delivery systems could increase the overcoming of the BBB by the use of drugs with a targeted-cell specificity modality. This approach permits the use of a lower dose of drug, a selective drug delivery to target tumor cells, both into the central core of the tumor and into the distal foci of tumor cells within areas often characterized by the integrity of the BBB [84,85]. This aspect is very important in early diagnosis, recurrences, preoperative histological and grade diagnosis, and preoperative treatment planning. Theranostic nanomedicine represents an integrated nanotherapeutic system, which can diagnose, deliver targeted therapy, and monitor the response to therapy. Nanomedicine also has the advantage of being able to target multiple tumor markers and deliver multiple agents simultaneously for synergy in addressing the challenges of cancer heterogeneity and adaptive resistance. Although nano-derived applications have great potential, there are some concerns about the nanoparticles’ possible adverse effects on human health and the environment. Nanotechnology is still a relatively young field, and little is known about the long-term effects of exposure to nanomaterials, especially in clearance organs such as the liver, spleen, and kidneys. Furthermore, the potential toxicity associated with the wide variety of nanomaterials available ranges from completely inert to highly toxic, which could slow their advancement into the clinic. Prior to the use of nanoengineered materials in clinical applications, major concerns, including biocompatibility and biodistribution, biosafety, side effects, and long-term effects, have to be addressed.

The recent discovery of many RNA molecules non-coding for protein, transcripted from DNA sequences different than II class genes (gene coding for protein) has drastically altered the traditional understanding of the human genome, genome-phenotype correlation, and gene-expression regulation. These nucleotidic sequences could easily move into the cell and among different cells to regulate gene expression and cellular phenotype. Moreover, new studies suggest a valid link between upregulated CSC markers with deregulated expression of miRNAs consistent with brain-seeking behavior of cancer cells. It is plausible that CSCs and miRNAs will play, in the near future, a large role in the molecular mechanism of tumorigenesis, including diagnosis, prognosis, and treatment.

Immunotherapy exhibits a great potential for prevention and treatment of brain cancers. Antigen-processing and presenting machinery components (APMs) play a crucial role in the generation of human leukocyte antigen (HLA) class I-TA peptide complex. Defective expression of APMs is a common phenomenon observed in a variety of human tumors [166]. The frequency of these defects is associated with clinical outcome, such as tumor progression and metastasis, as well as poor patient survival [167,168]. The results of a recent study indicate that low or defective expression of APM components β2-microglobulin, antigen processing-1 and calnexin, as well as paucity of CD8+ T cell infiltration in the primary breast cancers, may dictate high risks of developing brain metastasis. The authors suggest that metastases do not originate from a subclone of tumor cells that undergo downregulation of APM expression in the primary site, but rather, that an entire primary tumor with lower or defective expression of β2-microgloblin, TAP1 and calnexin might be more likely to spread to the brain [169].

Activated T cells and antibodies targeting tumor-associated antigens (TAAs) have been detected frequently in blood from patients with various types of tumor [170], supporting an active role for a host immune response against tumor [171]. It is now well accepted that tumors are able to evade detection and destruction by the immune system, even though many tumor types, especially melanoma, are capable of eliciting a strong immune response [172].

Ipilimumab is a first-in-class monoclonal antibody against cytotoxic T lymphocyte antigen-4 (CTLA-4), a molecule that prevents unwanted autoimmunity and establishes tolerance to self-antigens by downregulating T-cell activation via a homeostatic feedback loop. Ipilimumab has significant activity against stage IV melanoma with durable remissions in multiple phase 2 trials and now has demonstrated a significant survival benefit in a phase 3 randomized trial. In melanoma, it has shown marked benefits in patient populations previously refractory to treatment, patients with brain metastases, and patients who have progressed on prior systemic treatments. Evidence suggests that BRAF inhibition and immunotherapy may act synergistically. In preclinical studies, T-cell viability and function was preserved when peripheral blood mononuclear cells and BRAF^V600E^ mutant melanoma cells were exposed to clinically relevant concentrations of vemurafenib *in vitro* [173]. In addition, an analog of vemurafenib was shown to increase both antigen presentation by melanoma cells and their recognition by melanoma-specific T cells [174]. Together, these studies support the rationale that inhibition of BRAF^V600^ could render melanoma cells more susceptible to attack by immunotherapeutic strategies. The results of a preliminary, retrospective analysis suggest that it may be possible to determine the optimal sequence of treatments in patients with BRAF mutation-positive metastatic melanoma based on the presence of specific risk factors; however, further investigation in a larger number of patients is required to validate this hypothesis [175].

Further research on the concern the molecular mechanisms regulating the pathobiology of brain metastases should improve the understanding of the complex interactions between metastatic cells and the host environment. Therefore, improving our understanding of the molecular and cellular pathways involved in BM relapse during or after antiangiogenic and combination therapies will allow future therapeutic strategies to be tailored to each brain metastasis profile before, during, and after therapy. Detailed knowledge of cell biology and tumor biology are necessary to the rational design of new strategies (immune approaches, cancer stem cells, novel delivery systems, nanoparticles) for cancer and brain metastasis. On the basis of the data collected, and of the limits of the actual standard therapeutic protocol, we think that molecular-targeted therapy represents an interesting approach to modifying the metastatic process biology, by trying to modulate crucial pathways during the metastasis progression and more molecular targets of the same pathway or of two different pathways.

## Figures and Tables

**Table 1:** Metastatic suppressor genes involved in brain metastasis.

MSG	Chromosome location	Molecular alterations	Effect of the molecular alterations
KAI1 (CD82)	11p11.2	Mutations	Cell cycle control loss, proliferation
Nm23	17q21.3	Overexpression, amplification	Proliferation/invasion, cell transformation, cell cycle control
MKK4	17p12	Loss of heterozygosity, deletion, mutation	Proliferation, invasiveness, angiogenesis
CD44	11p11.2	Deletion, DNA hypermetilation	Proliferation, invasiveness
KISS-1	1q32.1	Mutations/deletion	Cell cycle control loss, Proliferation, chemiotaxis, invasion
SSeCKS	6q24-25.1	CDKN2/p16 deletion	Cell cycle control loss, proliferation
Brms1	11q13.2	Loss of heterozygosity	Regulating Akt/PKB signaling pathway loss; proliferation and tumor growth; invasiveness, angiogenesis
RhoGD12	12p12.3	Loss of heterozygosity	Pro-apoptotic action loss, proliferation
PTEN/MMAC1	10q23.3	Amplification, overexpression	Cell transformation, Proliferation, invasion

**Table 2:** Clinical trials * with antiangiogenic agents targeting brain metastasis. * Information obtained from the National Cancer Institute.

Drug	Target	Primary tumor	Notes
Trastuzumab	HER2	Breast Cancer	Phase I-II with Methotrexate and Carboplatin; Phase II with Everolimus and Vinorelbine
Neratinib (HK1-272)	HER2	Breast Cancer	Phase II
Lapatinib	HER2 EGFR	Breast Cancer, Lung Cancer	Phase II with WBRT; Phase II with Capecitabine
Afatinib	HER2 EGFR1	Breast Cancer	Phase II with or without Vinorelbine
Erlotinib	EGFR	Lung Adenocarcinoma, Non-Small Cell Lung Cancer	Phase II plus Pemetrexed; Phase II with or without WBRT; Phase III with or without WBRT and SRS
Icotinib	EGFR EGFR mutation	Non-Small Cell Lung Cancer	Phase III with WBRT, Phase II with WBRT, Phase I-II with WBRT, Phase II double dose
Sorafenib	VEGFR PDGFR	Breast Cancer	Phase I with WBRT; Phase II with SRS
Sunitinib	VEGFR, PDGFR	Kidney Cancer	Phase I with SRS; Phase II
Bevacizumab	VEGF	Breast Cancer	Phase I with WBRT; Phase II with Etoposide and Cisplatin
Dabrafenib	BRAFV600E	Melanoma	Phase II with SRS
Vemurafenib	BRAFV600E	Melanoma	Phase II
Everolimus	FKBP-12/mTOR	Breast Cancer	Phase II with Trastuzumab and Vinorelbine
RO4929097	NOTCH receptors	Breast Cancer	Phase I-II with WBRT/SRS
